# Biocontrol Potential of a Commercially Available Predator *Rhyzobius lophanthae* Blaisdell (Coleoptera: Coccinellidae) Against *Diaphorina citri* Kuwayama (Hemiptera: Liviidae)

**DOI:** 10.3390/insects16111083

**Published:** 2025-10-23

**Authors:** Gabriel Rodrigo Rugno, Jawwad A. Qureshi

**Affiliations:** Entomology and Nematology Department, Institute of Food and Agricultural Sciences (UF/IFAS), Southwest Florida Research and Education Center (SWFREC), University of Florida, 2685 State Road 29 North, Immokalee, FL 34142, USA

**Keywords:** Asian citrus psyllid, biological control, Huanglongbing (HLB), natural enemies, pest management

## Abstract

*Diaphorina citri* Kuwayama is a key pest of citrus and insect vector of Huanglongbing (HLB), also known as citrus greening disease, causing significant losses in Florida and other regions. The naturally occurring effective ladybeetle predators and their impact on *D. citri* reduced from years of insecticide use against this pest and are not available commercially. Additionally, most species are large-sized, while most eggs and neonates of *D. citri* are in hard-to-reach locations such as unopened leaves, which makes access difficult for them. We evaluated a commercially available small-sized predatory ladybeetle *Rhyzobius lophanthae* Blaisdell against *D. citri* immatures. A single adult consumed an average of 24.9 eggs and 8.7 first and second instar nymphs of *D. citri* within 24 h. Beetles exhibited Type II functional response against nymphs with an attack rate of 0.92 h^−1^ and a handling time of 0.08 h. Their consumption rate increased with nymphal density up to twenty per shoot. In the field test, beetles lived 10 days longer when confined with new shoots infested with *D. citri* immatures in a voile fabric sleeve cage in citrus trees every two days, versus seven days. In an open field release of *R. lophanthae* in a citrus orchard, these ladybeetles were found foraging in sentinel and neighboring trees infested with *D. citri*. The consumption rate of *R. lophanthae* on *D. citri* immatures and its survival in Florida orchards suggest its potential for biological control and Integrated Pest Management.

## Simple Summary

The Asian citrus psyllid (ACP) is a destructive pest of citrus in most parts of the world, warranting management because it vectors the causal pathogen of Huanglongbing (HLB), also known as citrus greening disease. Most predatory ladybeetles that provide biological control of ACP have been reduced due to the increased use of insecticides for several years and are not available commercially. ACP females deposit their eggs within the folds and crevices of young, unfurling “feather flush” leaves, where several eggs and neonates avoid contact with large predators. We evaluated a commercially available small-sized predatory ladybeetle *Rhyzobius lophanthae,* against ACP. This species effectively consumed eggs and young nymphs of ACP and exhibited a Type II functional response typical for effective ladybeetle predators. *Rhyzobius lophanthae* adults confined with infested shoots containing ACP immatures every two days in the citrus orchard lived 10 days longer than those confined every seven days. After open release in the citrus orchard, these ladybeetles were found foraging in sentinel trees where release was made and neighboring trees all infested with *D. citri*. Findings suggest the potential of *R. lophanthae* for biological control and Integrated Pest Management (IPM) in citrus orchards.

## 1. Introduction

*Diaphorina citri* Kuwayama (Hemiptera: Liviidae), commonly known as Asian citrus psyllid is a key pest of citrus crops and an insect vector of the “*Candidatus* (“*Ca*.”) Liberibacter asiaticus” (CLas), the pathogen responsible for causing Huanglongbing (HLB) or citrus greening disease, one of the most damaging diseases of citrus [1,2,3]. *Diaphorina citri* was identified in Florida in 1998 and encountered a strong response from several natural enemies already established or introduced, including ladybeetles [4,5,6,7]. A numerical response to an increase in the populations of a native ladybeetle, *Olla v-nigrum* Mulsant, was observed within a few years of the establishment of *D. citri* [8]. Additionally, a wide range of predatory insects were observed to feed on *D. citri* nymphs, including lacewings (Neuroptera: Chrysopidae), spiders (Araneae), and hoverflies (Diptera: Syrphidae) [4].

Biological control has been a key component of citrus pest management in Florida for decades and was also shown to be effective against the invasive *D. citri* [2,6,9]. A diet of *D. citri* nymphs was evaluated for ladybeetles *Curinus coeruleus* Mulsant, *Exochomus childreni* Mulsant, *Harmonia axyridis* Pallas, *O. v-nigrum*, *Cycloneda sanguinea* L., and *Coelophora inaequalis* (F.) and found suitable for most species [5]. Ladybeetles, such as *O. v-nigrum*, *H. axyridis*, *C. coeruleus*, and *C. sanguinea*, were found to significantly suppress populations of *D. citri* in southwest Florida [6]. Coccinellid beetles, including *H. axyridis, O. v-nigrum*, *C. sanguinea*, and *E. childreni*, were identified as key biological control agents of high-density *D. citri* populations in central Florida [10].

Considering the high reproductive rate of *D. citri* and its role as a vector of the pathogen responsible for causing HLB, its management requires the use of multiple control methods. The application of chemical control increased significantly in Florida’s commercial citrus production systems once HLB was identified in 2005, with growers using up to 12 spray applications per year to suppress *D. citri* populations [2,6,11,12,13]. These applications suppressed *D. citri* populations but also resulted in pest resistance to commonly used insecticides, increased production costs, and a significant reduction in the naturally occurring populations of natural enemies, which were shown to be significantly important in reducing the pest populations [12,14,15].

Ladybeetles are important natural enemies of insect and mite pests of multiple crops [6,16,17], and their reduction disrupts biological control in several agroecosystems. Unfortunately, ladybeetle species shown to be effective in suppressing *D. citri* populations have declined in number due to the increased use of insecticides and are not commercially available. There is a critical need to evaluate the effectiveness of commercially available species as potential predators for *D. citri* and candidates for augmentative biological control. In addition, most ladybeetle species found effective in the groves are large-sized, which makes it difficult for them to access the eggs and neonates of *D. citri* found within the folds and crevices of young, unfurling “feather flush” leaves [6].

*Rhyzobius lophanthae* Blaisdell (Coleoptera: Coccinellidae) is a small-sized ladybeetle species of Australian origin spread throughout the world, and it was introduced into California from New South Wales in 1889 and 1894 to control purple scale *Lepidosaphes beckii* Newman in citrus [18]. It has been introduced into North and South America, Europe, and Africa as a control agent for various armored scales [19,20]. Both larvae and adults of this species demonstrated voracious predatory behavior against diaspidid scales and consumed hundreds during their lifetime [21,22,23]. Since its introduction into California to control citrus scales, *R. lophanthae* has since spread and established in that state and is also available commercially as a biological control agent [21,24,25].

The effectiveness of *R. lophanthae* against scale pests is shown in some studies [26,27]; however, there is not much knowledge of its potential in providing biological control of *D. citri*, an insect pest vectoring HLB and devastating citrus industries in the world, including several states within the United States. *Rhyzobius lophanthae* was shown as a promising biological control agent for *D. citri* in California [28]. Considering that Florida is one of the largest citrus-producing states for the citrus juice industry and one of the most affected by HLB, with climatic conditions different than California, we investigated the potential of commercially available predator *R. lophanthae* against *D. citri* under laboratory and field conditions. We hypothesized that the *R. lophanthae* would significantly reduce the number of *D. citri* eggs and young nymphs under both laboratory and field conditions. Additionally, we expected that this predator’s functional response against *D. citri* would be comparable to that of other effective coccinellid biocontrol agents, and that its survival and dispersal after release in the cages and orchard would be positively affected by prey availability and release density.

## 2. Materials and Methods

Experiments were conducted at the Southwest Florida Research and Education Center (SWFREC) of the University of Florida-IFAS, Immokalee, FL, USA (Latitude: 26.484 N, Longitude: 81.435 W) and with a commercial grower in Labelle, FL, USA.

### 2.1. Maintenance of Rhyzobius lophanthae

*Rhyzobius lophanthae* adults used in this study were reared and shipped overnight to SWFREC in containers with honey as food from Foothill Agricultural Research, Inc. (550 West Foothill Parkway, Corona, CA 92882, USA). We maintained beetles in a climate-controlled room at a temperature of 25 ± 2 °C, relative humidity of 70 ± 10%, and a photoperiod of 14 L:10 D h. Experiments were conducted 24 h after receiving the insects.

### 2.2. Maintenance of Diaphorina citri Colony and Field Populations

A colony of *D. citri* was maintained on orange jasmine *Murraya paniculata* (L.) at the SWFREC, Immokalee, FL. The potted plants of *M. paniculata* were kept in a screen house covered with “Antivirus” insect netting to prevent pest infestations. *Murraya paniculata* plants containing young shoots suitable for oviposition by *D. citri* were placed in the insect-proof cages (45 × 45 × 50 cm) (BioQuip, Rancho Dominguez, CA, USA) and exposed to *D. citri* adults from the HLB negative colony. Shoots infested with eggs and nymphs of *D. citri* were removed from the plants as needed to evaluate *R. lophanthae* in the experiments. Field experiments were conducted using shoots infested with feral populations of *D. citri* in citrus trees developing under natural conditions in an orchard planted with “Valencia” sweet orange on swingle rootstock in southwest Florida, where most trees and psyllids are infected with HLB.

### 2.3. Mortality of D. citri Eggs and Nymphs Exposed to R. lophanthae

Shoots of *M. paniculata* (30 cm high) infested with *D. citri* eggs from the colony maintained at SWFREC were used in the experiments. To prevent desiccation during the experiment, shoots were individually placed in a Falcon™ vial (a 50 mL polypropylene centrifugal tube, 116 × 29 mm, Thermo Fisher Scientific Inc., Waltham, MA, USA) containing water. A thermoplastic film (Parafilm^®^, Thomas Scientific, Swedesboro, NJ, USA) was used to cover the open end of the vial and secure the shoot, protecting nymphs from crawling into the water. To measure the beetle’s impact on *D. citri* eggs, the number of eggs on the shoot was counted using a stereo microscope. Egg numbers were standardized to 80 per shoot for one adult of *R. lophanthae*. Beetles were starved for 12 h prior to the experiment. Each shoot with *D. citri* eggs was enclosed with one *R. lophanthae* adult in a voile fabric sleeve cage (5 × 7 inches). A shoot of *M. paniculata* infested with *D. citri* eggs with no *R. lophanthae* was used as a control. There were 20 randomly assigned replicates for the beetle treatment and control.

In the experiment to test *R. lophanthae* against *D. citri* nymphs, a mixed population of first- and second-instar *D. citri* nymphs was used from the colony maintained at SWFREC. The experimental procedure was the same as for eggs, except that 26 nymphs per shoot were confined with a single adult of *R. lophanthae*. There were 20 randomly assigned replicates of nymph-infested shoots confined with beetle adults, and a control containing an infested shoot without beetle. The experiments were conducted in a climate-controlled room under conditions described for the *R. lophanthae* colony.

In the experiments with eggs and nymphs, we evaluated the mortality 24 h after confinement. We removed the adults of *R. lophanthae* from cages containing shoots infested with eggs, nymphs, and beetles, and examined the shoots under a stereo microscope to determine the number of alive and dead individuals. Eggs and nymphs that were intact were considered alive, and those that were deformed or not found were considered dead. The nymphs that did not move after being probed with a soft hairbrush were also considered dead. This was performed because a few dead nymphs were observed in the control.

### 2.4. Predation Capacity of R. lophanthae on D. citri Nymphs

*Rhyzobius lophanthae* adults were tested at six densities of first-instar *D. citri*. Nymphs at a density of 1, 5, 10, 15, 20, and 40 per shoot were provided to individual adults of *R. lophanthae* in each of these six density treatments. There was a respective control for each density treatment, which consisted of shoots infested with nymphs without *R. lophanthae*. The cut ends of the orange jasmine shoots were placed in Falcon™ vial (a 50 mL centrifugal tube, 116 × 29 mm) filled with water to prevent desiccation. We used a voile fabric sleeve cage (5 × 7 inches) to confine the shoots with or without beetles. Beetles were starved for 12 h before testing in the experiment. There were twenty replicates of each density treatment, and each replicate consisted of an infested shoot with nymphs and a beetle. There were 20 replicates for each density with or without the beetle. The experimental units were maintained in a climate-controlled room at a temperature of 25 ± 2 °C, a relative humidity of 70 ± 10%, and a photoperiod of 14 L:10 D h. We evaluated the mortality of *D. citri* nymphs 24 h following confinement in the sleeve cages using the criteria described in the previous experiment.

### 2.5. Field Evaluation of the Release Rates of R. lophanthae Adults on D. citri Immatures

This study was conducted in a citrus orchard of five-year-old Valencia orange trees planted at a density of 500 trees per acre at SWFREC. Pesticides have historically been used to control pests in this block; however, the last application was four months prior to the start of this experiment. Therefore, we did not expect any residual effects of the chemicals. *Rhyzobius lophanthae* adults were confined with shoots that contained a mixed population of 40 *D. citri* eggs and nymphs (20 eggs and 20 first instars) in a voile fabric sleeve cage (5 × 7 inches). Three rates of *R. lophanthae*: one, three, and five adults per shoot, were tested. Beetles were starved for 12 h prior to the experiment. The control consisted of shoots infested with the same number of *D. citri* eggs and nymphs without beetles. A total of 20 shoots were used in each density treatment. A completely randomized design was used for the experiment. All shoots were examined with a 14× magnifying lens to count and standardize the number of eggs and nymphs per shoot. The experimental units were marked and then randomly assigned to the treatments. Following 24 h of confinement, shoots with sleeve cages were collected from the trees and brought to the laboratory, where the number of *D. citri* individuals was counted under a stereoscopic microscope.

### 2.6. Field Evaluation of the R. lophanthae Adult Survival at Two Densities of D. citri Immatures

These tests were conducted considering that *D. citri* populations are associated with the production of new shoots in the trees, which the adults need to reproduce and the nymphs to develop. The mature citrus trees produce most new shoots during the spring and sporadic growth during the other seasons [7,29,30]. The situations of reduced or no availability of new shoots and *D. citri* immatures could negatively impact the survival of the natural enemies. The survival of *R. lophanthae* adults was evaluated by simulating two scenarios of *D. citri* immature availability to adult beetles. A single adult of *R. lophanthae* was confined with a shoot infested with 20 *D. citri* (eggs and first instars) in a voile fabric sleeve cage (5 × 7 inches) in two treatments. Beetles were transferred to a new infested shoot with the same amount of *D. citri* every two or seven days, constituting two treatments. There were 30 replicates of each treatment in a completely randomized design. Mortality of *R. lophanthae* in the treatments was evaluated every 24 h. This experiment was conducted in the same block of citrus in which the suppression of *D. citri* was evaluated under three different rates of *R. lophanthae*.

### 2.7. Release of R. lophanthae Adults in a Citrus Orchard

*Rhyzobius lophanthae* adults release and recovery were evaluated in a 15-acre orchard of a mature Valencia sweet orange on Swingle rootstock in LaBelle, Florida (26°42′16.3″ N 81°27′09.7″ W). The trees were over 20 years old, and the orchard was divided into four replicates to include five pest management programs, which were: (1) conventional insecticides (CI) and biological control (BC); (2) CI, organic insecticides (OI) and BC; (3) OI, horticultural mineral oil (HMO) and BC; (4) HMO only; (5) BC only. These programs were implemented over a two-year period and continued until two months prior to the release and evaluation of *R. lophanthae*. Beetles were released in all four plots (replicates) of each pest management program. There were approximately 60 trees in each plot, and release was made in the center of the plot. A container containing 500 ladybeetles was hung from a citrus tree at a height of 150 cm, and then the lid was removed. The presence of beetles in the trees was evaluated using the stem tap method [31] and the suction sampling method [32]. We sampled nine trees using the tap sampling method in the vicinity of the tree where a release was made. Suction sampling was conducted using a modified vacuum, and five trees in the vicinity of the release sites were sampled for one minute per tree. A pre-sample using tap and suction sampling was conducted, which revealed that no *R. lophanthae* were already present in the field. The vacuum sampling was conducted two days after the release, and the tap sampling six days after the release. In the tap sample, the ladybeetles were counted as they fell onto the sheet, whereas in the vacuum sample, the samples were brought to the laboratory, where the ladybeetles were counted.

### 2.8. Statistical Analysis

A *t*-test was performed to compare the average mortality of *D. citri* eggs and nymphs in the presence and absence of *R. lophanthae*. Assumptions of normality and homogeneity of variance were met, and analyses were conducted on untransformed data. We used a generalized linear model (GLM) [33] and a binomial distribution to analyze the percentage of psyllid suppression in the field, while a Poisson distribution was used to analyze the tap and vacuum sample data. These data were evaluated using a half-normal graph with a simulated envelope [34]. To evaluate the suppression of *D. citri* in the field, in addition to the GLM of the binomial type, multiple comparisons (Tukey’s test, *p* < 0.05) were performed using the glht function of the multicomp package with *p*-value adjustment. All analyses were performed using the statistical program “R” version 4.1.2 (R Core Team 2021).

The analyses for functional responses modeling were carried out using the ‘frair’ package (version 0.5.100) [35]. The type of functional response was determined by the linear and quadratic coefficients. For prey density experiments, a significantly negative linear coefficient and a type II functional response were observed for *R. lophanthae*, which indicates the proportion of prey consumed declines with the initial prey density [36]. The function ‘frair fit’ from the R package ‘frair’ [35,37] was used to obtain attack rate (α) and handling time (T_h_). Rogers’ random predator Equation (2) [38] was used to model the relationship between the number of prey consumed (Ne) and initial prey density (N_0_):

Ne = N_0_ [1 − exp(αT_h_ N_e_ − αT)]

where Ne is the number of prey consumed, N_0_ is the initial prey density, α is the attack rate, T is the total exposure time (1 day), and T_h_ is the handling time. To visualize the uncertainty around the fitted FRs, bootstrapping (*n* = 1500) was used to construct 95% confidence intervals.

Survival analysis was performed using the R package (version 3.2-13). Kaplan–Meier survival curves were plotted, and differences in survival rates between treatments were calculated using the log-rank test and Holm-corrected *p*-values. All analyses were carried out in R (version 4.1.2 (R Core Team 2021)).

## 3. Results

### 3.1. Rhyzobius lophanthae Predation of D. citri Eggs and Nymphs

**Figure 1:**
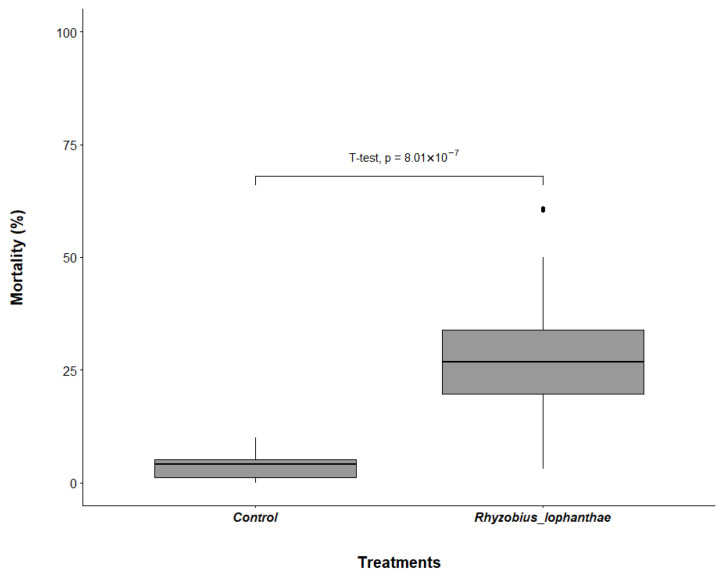
Mortality of *Diaphorina citri* eggs (%) (±SE) in the presence and absence of *Rhyzobius lophanthae* adults over a 24 h period in laboratory conditions.

**Figure 2:**
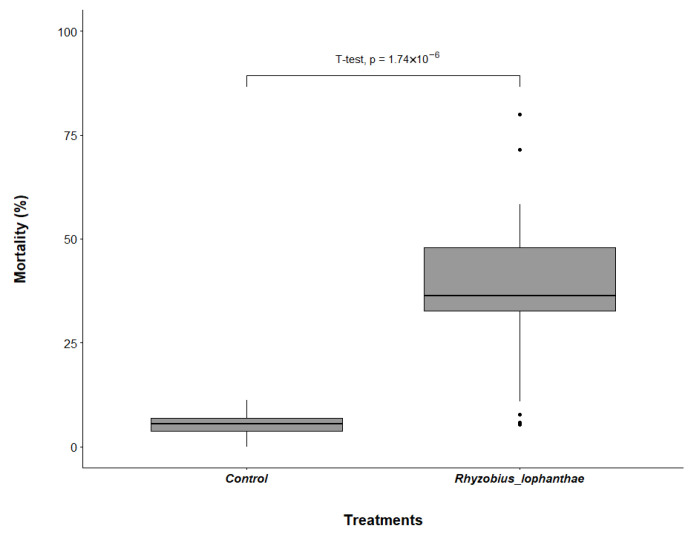
Mortality of *Diaphorina citri* nymphs (%) (±SE) in the presence and absence of *Rhyzobius lophanthae* adults over a 24 h period in laboratory conditions.

*Rhyzobius lophanthae* confined with eggs of *D. citri* on the shoots of orange jasmine in the laboratory provided a significant reduction in eggs 24 h after confinement in comparison with the control (df = 38, t = 7.0098, *p* < 0.001) (Figure 1). The average egg mortality in treatment containing *R. lophanthae* was 28.6% (24.9 ± 4.5 eggs), compared with 3.8% (2.7 ± 0.5 eggs) in the control. *Rhyzobius lophanthae* adults were also effective in providing a significant reduction in *D. citri* nymphs in comparison with the control (df = 36, t = 6.7228, *p* < 0.001) (Figure 2). There was an average mortality rate of 35.6% (8.7 ± 1.1 nymphs) for those exposed to *R. lophanthae*, and 5.2% (1.2 ± 0.2 nymphs) in the control.

### 3.2. Rhyzobius lophanthae Predation Capacity at Different Densities of D. citri Immatures

**Figure 3:**
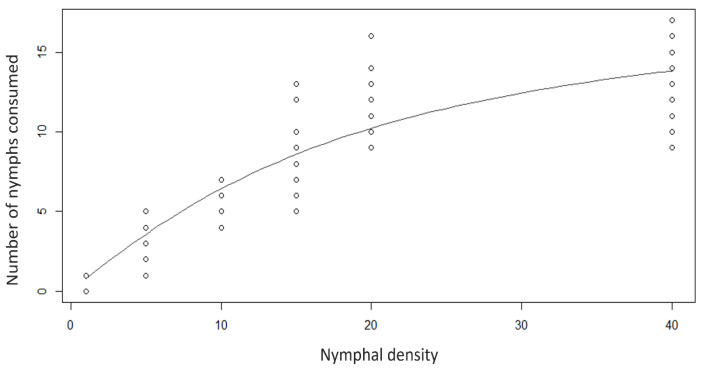
The number of *Diaphorina citri* nymphs consumed by *Rhyzobius lophanthae* adults within 24 h. The curve represents the best-fitting functional response model (flexible type II functional response); 95% bootstrapped confidence intervals.

One adult of *R. lophanthae* was able to consume an average of 0.8 ± 0.09 (80.0%), 3.4 ± 0.25 (68.0%), 5.75 ± 0.24 (57.5%), 8.95 ± 0.53 (59.7%), 11.65 ± 0.42 (58.2%) and 12.75 ± 0.47 (31.9%) nymphs, respectively, when confined with infested shoots at densities of 1, 5, 10, 15, 20, and 40 nymphs per shoot. The increase in predation on *D. citri* nymphs was evident from the lowest to the highest density (Figure 3). The regression analyses suggested that this predator at the adult stage demonstrated a Type II functional response. The average time spent on processing prey or handling time was 0.08 h (0.07–0.09), and the attack rate was 0.92 h^−1^ (0.77–1.10).

### 3.3. Diaphorina citri Suppression at Three Release Rates of R. lophanthae

**Figure 4:**
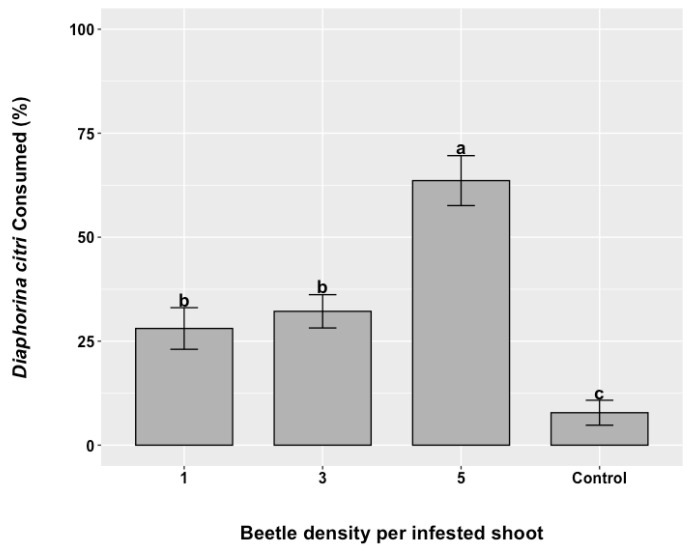
The mortality percentage (±SE) of *Diaphorina citri* immatures under 1, 3, and 5 *Rhyzobius lophanthae* adults per infested shoot. Different letters on the columns indicate a significant difference between treatments (GLM with binomial distribution followed by post hoc Tukey test, *p*  <  0.05).

*Rhyzobius lophanthae* adults confined at different rates on citrus trees with infested shoots containing *D. citri* eggs and first instars provided a significant reduction compared to the control (df 3, 69, F = 17.827, *p* < 0.001). At 1, 3, and 5 beetles per infested shoot containing a mixed population of eggs and nymphs of *D. citri*, a percentage reduction of 28.05 ± 6.11, 32.17 ± 4.04, and 63.60 ± 5.69, respectively, was observed in psyllid populations. The greatest reduction was seen at 5 beetles per infested shoot, whereas the level of predation was half as high in shoots with one or three ladybeetles, with no significant difference between these two rates (Figure 4).

### 3.4. Rhyzobius lophanthae Survival at Two Levels of Diaphorina citri Populations, and Recovery from an Open Release in a Citrus Orchard

**Figure 5:**
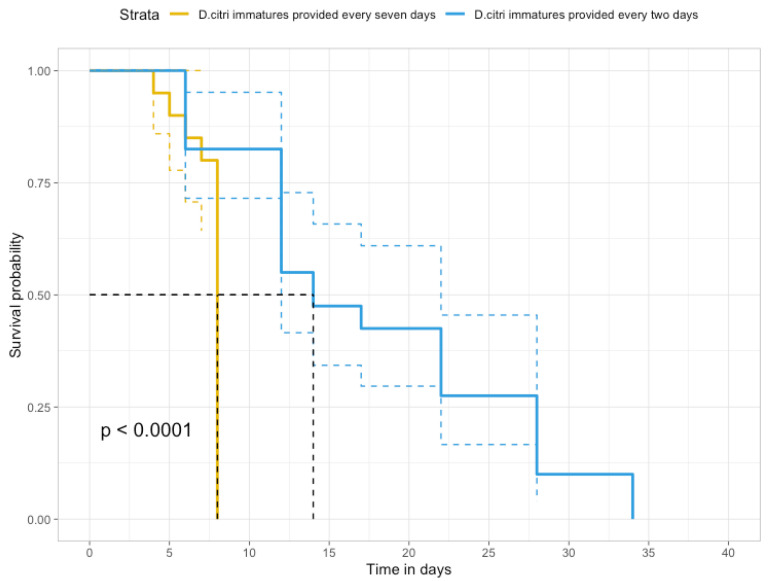
The proportion of *Rhyzobius lophanthae* adults that survived in the field when supplied with *D. citri* every two and seven days. There was a significant difference in the survival curves of the two treatments (*p* < 0.0001).

*Rhyzobius lophanthae* adults that had access to new shoots infested with *D. citri* immatures every two days in the field lived significantly longer than the adults to which infested shoots were made accessible every seven days (Figure 5). The half-life of the beetles with access to shoots infested with *D. citri* immatures at two and seven days in the field was 17.85 ± 1.45 and 7.50 ± 0.26, respectively.

There was no difference in the number of *R. lophanthae* recovered after open release in the citrus orchard between the five program areas using tap samples (df 4, 15, F = 0.4559, *p* = 0.7668) and vacuum samples (df 4, 20, F = 0.1678, *p* = 0.9523). The average number of specimens collected per plot ranged from 12 to 15 for tap samples and 11 to 15 for vacuum samples.

## 4. Discussion

Our study demonstrated the significant potential of commercially available *R. lophanthae* adults to prey on and suppress *D. citri*, currently the most significant pest of citrus crops in citrus-producing regions worldwide. The findings of our study showed that *R. lophanthae* adults consumed and reduced the number of *D. citri* eggs and small nymphs provided to them through infested shoots, compared to shoots without beetles, under Florida conditions. The results of another study demonstrated that, in addition to adults, the larvae of *R. lophanthae* also feeds on eggs and nymphs of *D. citri* in a no-choice scenario [28]. *Rhyzobius lophanthae* is a small-sized beetle, and its consumption of eggs and young nymphs of *D. citri* in young shoots is a significant contribution, considering that most of these early young stages are generally hidden in the unfolded leaves of newly developing buds and shoots and are not easily accessible to large predators. There are not many small-sized ladybeetles abundant in the citrus orchards, nor are the studies demonstrating their effectiveness in suppressing *D. citri*. *Exochomus childreni* Mulsant, a naturally occurring small-sized beetle, was shown to perform well on the diet of *D. citri* immatures; however, its occurrence in citrus orchards in Florida, particularly in the southwest region, is rare [5,6]. The larvae and adults of ladybeetles, shown to be significant contributors to *D. citri* mortality in citrus orchards, such as *O. v-nigrum*, *H. axyridis*, *C. coeruleus*, and *C. sanguinea,* are large, and it is not easy for them to access eggs and small nymphs in hard-to-reach places such as newly opening buds and shoots [6].

The type II functional response indicates predator suppression of a pest population, especially in the early stages of an outbreak or establishment and has been observed in several ladybeetles against various prey species [35,36,37,38], which is significant for their effectiveness as biological control agents [39]. This type of response suggests that with an increase in prey density, the consumption rate by ladybeetles initially increases and eventually levels off at a maximum consumption and satiation. *Rhyzobius lophanthae* exhibited a type II functional response in its consumption of *D. citri* immatures. Its consumption rate increased with increasing density of the prey and tended to stabilize at 40 immatures per shoot. Huang et al. observed a type II functional response for different development stages of *H. axyridis* (Coleoptera: Coccinellidae), a large-sized ladybeetle that preys on *D. citri,* and compared their results with those obtained for *R. lophanthae*; they observed a similar attack rate and a higher handling time [40]. The attack rate and handling time of *Hippodamia variegata* (Goeze) (Coleoptera: Coccinellidae), another large-sized ladybeetle, on *Aphis gossypii* Glover (Hemiptera: Aphididae), were comparable to those in our study [41]. The similarity in the attack rate and handling time of *R. lophanthae* and large-sized ladybeetles is encouraging, considering that the latter have already been shown to be effective in suppressing *D. citri* in the field [6]. These indicators of the overall magnitude of the functional response of a predator are likely to vary under different situations, such as prey type and availability, predator life stage and abundance, temperature, and experimental arena [42,43,44].

*Diaphorina citri* suppression doubled by increasing the beetle density to five per infested shoot compared to 1 or 3 beetles per shoot; however, there was no difference in the effectiveness of the ladybeetle between these two latter densities. Apparently, there was no intraspecific competition between the predators when more than one beetle was attacking prey on an infested shoot. Huang et al. observed a reduction in *D. citri* suppression with an increase in the number of beetles in the experimental arena, which was attributed to competition [40]. In the field, high levels of ladybeetle populations, such as five per shoot, are not common, unless orchards are inundated with ladybeetles and a few infested shoots are available. Although two to three individuals of the same or two ladybeetle species feeding on an infested shoot have been observed in some instances. However, the consumption of eggs and nymphs of *D. citri* by *R. lophanthae*, as well as the reduction in both life stages by individual adults of the ladybeetle, reflects highly on their potential use as a biological agent of *D. citri*.

*Rhyzobius lophanthae* lived for weeks under Florida conditions when it preyed solely on *D. citri* eggs and nymphs, and its survival was only dependent on the availability of the psyllid diet. Beetles that had access to *D. citri*-infested shoots every two days lived longer than those that had access to them after seven days. This suggests that these beetles can effectively utilize the psyllid diet for their survival during a period when young shoots and psyllid infestations are not common. *Diaphorina citri* requires young shoots to develop and reproduce. The mature citrus trees in Florida produce many of their young shoots in spring, followed by sporadic production in summer and fall [7,29]. Thus, more psyllid eggs and immatures are available for predators in spring than in summer and fall. Additionally, citrus trees are colonized by many other pests such as scales, aphids, mites, and thrips, which generalist predators can utilize to support their existence in the orchards. For example, different species of aphids are found during summer and fall. *Rhyzobius lophanthae* adults were able to disperse from the release trees to the neighboring trees in the citrus orchard. The released adults were captured in the trees where releases were made and in the neighboring trees up to six days post-release. Beetles were observed foraging in many parts of the citrus trees a couple of hours after release, actively searching for prey, including the young shoots that the *D. citri* prefers for feeding and reproduction.

To improve the effectiveness of *D. citri* management and reduce the imbalance in natural biological control caused by insecticide use, additional biological control agents should be integrated into the citrus agroecosystem. Many studies have focused on *Tamarixia radiata* Waterston (Hymenoptera: Eulophidae), a parasitoid of *D. citri*, but very few have examined the potential of new predators to control *D. citri* [7,45,46,47]. Findings from our study suggest that *R. lophanthae* is capable of preying on eggs and young nymphs of *D. citri* in citrus trees under Florida conditions. At least 50% of the adults that were rotated every two days to new shoots infested with psyllid immatures in sleeve cages on citrus trees in the orchard lived for about two weeks, while 75% of those that were provided with infested shoots every seven days survived for one week. Those released in the citrus orchard were observed foraging for prey within a week of release. This study provides valuable insights into the *R. lophanthae* consumption of *D. citri* and its survival on the psyllid diet under Florida conditions. The addition of commercially available predators may strengthen the existing predatory guild in citrus orchards and counteract the reduction in natural enemies and their impact from extensive use of chemical control. The small size of *R. lophanthae* makes it valuable in targeting the eggs and younger nymphs of *D. citri*, most of which are protected inside the unfolded leaves and places where contact with large predators and even insecticide sprays is reduced. However, further research is warranted to investigate the addition of *R. lophanthae* in integrated citrus pest management, including its establishment in citrus orchards, interaction with existing species, frequency, and rate of release.

## Data Availability

The original contributions presented in this study are included in the article. Further inquiries can be directed to the corresponding author.
